# AGE-FRIENDLY TRANSFORMATION: AN EXAMINATION OF COMMUNITY MECHANISMS INFLUENCING INVOLVEMENT

**DOI:** 10.1093/geroni/igac059.290

**Published:** 2022-12-20

**Authors:** Caitlin Coyle, Ceara Somerville

**Affiliations:** University of Massachusetts Boston, Boston, Massachusetts, United States; University of Massachusetts Boston, Boston, Massachusetts, United States

## Abstract

Age-friendly community initiatives (AFCIs) have become key policy efforts aimed at improving quality of life for older residents, but there is limited evidence about the process. This mixed methods study draws on survey and demographic data from 350 municipalities in Massachusetts to characterize communities by these categories: 1) not interested in AFCIs (n=109); 2) interested in learning more about AFCIs (n=84); 3) planning for age-friendly action (n=71); and 4) maintaining an AFCI (n=86). Interview data from key-informants contextualize the process of developing an AFCI. Thematic analyses suggest that progression through AFCIs is self-defined by the accumulation of momentum. Communities committed to AFCIs have higher proportions of vulnerable residents (e.g., living with disability, living alone, non-English speaking). Municipal resources (e.g., budget, aging services) correlate with more advanced stages of AFCIs. Implications of the variability across AFCIs, including the effort required for moving from concept to execution of AFCIs, will be discussed.

